# Using the CDC’s Worksite Health ScoreCard as a Framework to Examine Worksite Health Promotion and Physical Activity

**DOI:** 10.5888/pcd15.170463

**Published:** 2018-06-21

**Authors:** Leah K. Gutermuth, Erin R. Hager, Keshia Pollack Porter

**Affiliations:** 1 Department of Pediatrics, Growth and Nutrition Division, University of Maryland School of Medicine, Baltimore, Maryland; 2 Department of Health Policy and Management, Johns Hopkins Bloomberg School of Public Health, Baltimore, Maryland

## Abstract

**Introduction:**

Worksite health promotion programs are emerging as an effective approach for addressing the adult obesity epidemic and improving the overall health of employees.

**Methods:**

We conducted a scoping review to identify articles that described a physical activity component (eg, promoted increased physical or reduced sitting time) of a worksite health promotion intervention. Our search specified full-length articles published in English from January 2000 through July 2015. We used the Centers for Disease Control and Prevention’s Worksite Health ScoreCard, a validated tool, as a framework to summarize information on organizational supports strategies (18 questions) and physical activity strategies (9 questions) implemented by worksite health promotion programs. We also determined whether or not the included studies reported significant (*P* < .05) improvements in physical activity.

**Results:**

We identified 18 worksite health promotion programs; 11 produced significant improvements in physical activity. Incentives, health risk assessments, health promotion committees, leadership support, marketing, and subsidies or discounts for use of exercise facilities were the most effective organizational supports strategies cited, and physical activity seminars, classes, and workshops were the most effective physical activity strategies cited.

**Conclusion:**

The use of the Health ScoreCard allowed for a practical interpretation of our findings, which can inform next steps for the field. Future research should explore the relationships between components of worksite health promotion programs and their outcomes to further develop best practices that can improve worker health and promote physical activity.

## Introduction

Worksite health promotion programs are an effective approach for improving the overall health of working adults in the United States (1). Nearly 38% of American adults are obese (2). Because the average employee spends 7.6 hours per day at work (3), the workplace is an important place in which to influence the behavioral and environmental determinants of obesity (4). Worksite health promotion programs that focus on improving nutrition, physical activity, or both have produced reductions in weight and body mass index (5). Worksite health promotion programs may be a powerful, cost-effective strategy to prevent obesity and promote health among US adults (6–10).

The Centers for Disease Control and Prevention’s (CDC’s) Worksite Health ScoreCard (HSC) is a tool for employers to assess the implementation of evidence-based worksite health promotion interventions (11). The HSC consists of 16 components that each include a list of evidence-based strategies. It has been used by state health departments, worksites, and their partners as a tool to expand implementation of evidence-based worksite practices, assist with creating sustainable programs, assess implementation, evaluate environmental changes, and track program components (12).

This review had 2 primary objectives: 1) to use the HSC as an evidence-based framework to examine physical activity strategies of worksite health promotion programs, and 2) to compare the frequency of using selected worksite health promotion strategies (included in the HSC) among programs that reported improving physical activity among employees. 

## Methods

We conducted a scoping review in August through October 2015. A scoping review is a synthesis of the literature, typically conducted as a preliminary investigation to identify the range and nature of existing research (13). Scoping reviews provide insight into gaps in research (13). Our review aimed to identify articles that described a physical activity component (eg, promoted increased physical or reduced sitting time) of a worksite health promotion intervention and to determine which strategies were most effective.

We identified articles by using the OneSearch engine (www.hshsl.umaryland.edu). This search engine provides an access point to 60 databases; a single search on OneSearch can identify articles from various search engines. We used the following keywords to find peer-reviewed articles that were published from January 2000 through July 2015: “worksite health promotion” or “employee health” and “physical activity.” To further identify potential articles, we added the search terms “organizational support” or “policy.” We reviewed titles and abstracts for studies that focused on improving physical activity behaviors. To supplement the search, we examined reference lists from relevant articles. The article selection was driven by a logical approach that partly reflected our own opinion and expertise in the field of worksite health promotion. We did not limit the search to specific study designs, outcomes, or worksite characteristics (eg, sex or age of participants; size, setting, number of employees; location of worksite).

### Study selection

After an initial search, we reviewed titles and abstracts to determine articles that met inclusion criteria. The following inclusion criteria were used: the intervention took place in a workplace, the program included components that involved physical activity, and the study was published in English. We excluded any articles that did not provide enough details about the program components to analyze by using the HSC. Only full-length articles were considered. After an initial screening of the articles, we selected 18 for review; they were published from 2002 to 2015. Because we did not limit the types of studies designs that were included for this review, we did not systematically evaluate the quality of the evidence.

### Data extraction

The HSC covers 16 topics (eg, tobacco control, nutrition, stress management, physical activity, organizational supports). We focused on the topics of physical activity and organizational supports (Table 1). These were selected because physical activity has stronger benefits for disease prevention, weight control, mental health, stress management, and productivity compared with other topics of the HSC (14,15), and because a perception of organizational commitment is associated with physical activity outcomes (16–18). CDC defines each topic with a set of questions, or strategies (18 for organization supports and 9 for physical activity). We selected these strategies because worksite physical activity that incorporates environmental support, coaching, and a combination of physical activity components has potentially positive effects on employee weight outcomes (19–22).

**Table 1:** Organizational Supports Components and Physical Activity Components of CDC’s Health ScoreCard^a^

Component	Yes, No. of Points	No, No. of Points
**Organizational supports: During the past 12 months, did your worksite . . .**		
Conduct an employee needs and interests assessment for planning health promotion activities?	1	0
Conduct employee health risk appraisals/assessments through vendors, on-site staff, or health plans and provide individual feedback plus health education?	3	0
Demonstrate organizational commitment and support of worksite health promotion at all levels of management?	2	0
Use and combine incentives with other strategies to increase participation in health promotion programs?	2	0
Use competitions when combined with additional interventions to support employees making behavior changes?	2	0
Promote and market health promotion programs to employees?	1	0
Use examples of employees role modeling appropriate health behaviors or employee health-related “success stories” in the marketing materials?	1	0
Tailor some health promotion programs and education materials to the language, literacy levels, culture, or readiness to change of various segments of the workforce?	3	0
Have an active health promotion committee?	2	0
Have a paid health promotion coordinator whose job (either part-time or full-time) is to implement a worksite health promotion program?	2	0
Have a champion(s) who is a strong advocate for the health promotion program?	2	0
Have an annual budget or receive dedicated funding for health promotion programs?	2	0
Set annual organizational objectives for health promotion?	2	0
Include references to improving or maintaining employee health in the business objectives or organizational mission statement?	1	0
Conduct ongoing evaluations of health promotion programming that use multiple data sources?	2	0
Make any health promotion programs available to family members?	1	0
Provide flexible work scheduling policies?	2	0
Engage in other health initiatives throughout the community and support employee participation and volunteer efforts?	2	0
**Your worksite’s organizational supports section score (total points possible: 33)**		
**Physical activity: During the past 12 months, did your worksite . . . **		
Provide an exercise facility on-site?	3	0
Subsidize or discount the cost of on-site or off-site exercise facilities?	3	0
Provide environmental supports for recreation or physical activity?	3	0
Post signs at elevators, stairwell entrances or exits and other key locations that encourage employees to use the stairs?	3	0
Provide organized individual or group physical activity programs for employees (other than the use of an exercise facility)?	3	0
Provide brochures, videos, posters, pamphlets, newsletters, or other written or online information that address the benefits of physical activity?	1	0
Provide a series of educational seminars, workshops, or classes on physical activity?	2	0
Provide or subsidize physical fitness assessments, follow-up counseling, and physical activity recommendations either on-site or through a community exercise facility?	3	0
Provide free or subsidized self-management programs for physical activity?	3	0
**Your worksite’s physical activity section score (total points possible: 24)**		

a Adapted from the Centers for Disease Control and Prevention’s Worksite Health ScoreCard (11).

The HSC assigns point values of 1, 2, or 3 (1 = good, 2 = better, and 3 = best) to indicate the level of effect each strategy has on the associated health topic or outcome and the strength of evidence for this effect (eg, brochures = 1 point, lifestyle counseling = 3 points). Among the 18 organizational supports strategies included in the HSC, 5 are categorized as good, 11 as better, and 2 as best. Among the 9 physical activity strategies, one is categorized as good, one as better, and 7 as best.

The lead author (L.K.P.) searched for strategies listed in the HSC and extracted information on these strategies from the articles. The lead author then sent a random sample of articles for abstraction to the 2 coauthors. This process revealed consistency in abstraction and validated the abstraction conducted by the lead author. We assessed 25 of the 27 HSC organizational supports and physical activity strategies. We chose not to extract information on 2 HSC strategies described by the following questions: “During the past 12 months, did your worksite ‘have an annual budget or receive dedicated funding for health promotion programs?” (better) and “provide free or subsidized self-management programs for physical activity?” (best). We did not extract this information because all the included studies had a physical activity program (a criterion for inclusion) and all had some type of funding, which meant that these measures would not vary. We included the strategy described by the question “During the past 12 months, did your worksite conduct ongoing evaluations of health promotion programming that use multiple data sources?,” but all studies confirmed multiple data sources.

We reviewed and assessed articles by using the HSC as a framework for evaluating the relevant organizational supports and physical activity strategies of each program. The lead author (L.K.P.) used the HSC strategies as a checklist for each article. After creating the side-by-side comparison of HSC strategies by study, we then determined whether the studies reported significant (*P* < .05) improvements in physical activity.

## Results

Eighteen articles were identified, and all included an intervention; however, the study designs varied. Ten studies used randomization (23–32), including wait-list control groups (24,25,31), random allocation (23,27–30,32), and a crossover design (26). Among the 8 remaining studies, 5 used a single-group pre–post design (33–37), 1 employed a non-randomized controlled trial (38), 1 used a non-randomized interrupted-time-series approach (39), and 1 used a quasi-experimental design (40). Five of the studies were pilot studies (26,34,36,37,40), and 4 were feasibility studies (24,29,32,35) (Table 2).

**Table 2:** Summary of Worksite Health Promotion Programs Identified in a Scoping Review That Used the CDC’s Worksite Health ScoreCard^a^ as a Framework to Examine Worksite Health Promotion and Physical Activity

Author, Year	Study Design	Single or Multiple Worksites	Target Behavior or Disease	Sample Population	Significantly Improved Physical Activity^b^	Type of Physical Activity Improvement
Aldana et al (23), 2005	4-week RCT with untreated control group	Single	Physical activity and diet	Rockford, Illinois (n = 145)	Yes	Total steps per week
Blake et al (33) (2013)	5-year ecological study	Single	Physical activity, well-being (general health and mood), stress and diet	National Health Services employees in the United Kingdom (n=1,134)	Yes	Activity at work, active travel to work, walking or cycling for at least 10 min, meeting physical activity guidelines
Jules Pretty et al (24) (2014)	8-week parallel-group randomized controlled trial	Multiple	Physical activity	Desk-based employees of the *Financial Times* and London Stock Exchange in the United Kingdom (n = 73)	No	None
Campbell et al (25) (2002)	18-month randomized experimental design	Multiple	Physical activity, diet, smoking, and cancer screening	Rural, blue-collar women working in eastern North Carolina (n = 538)	Yes	Stretching and flexibility exercises
Chae et al (34) (2015)	8-week single-group pretest–posttest pilot study	Single	Physical activity	Sedentary office workers from an airline company in Seoul, South Korea (n = 70)	Yes	Daily steps
Chau et al (26) (2014)	4-week randomized controlled trial pilot design	Single	Physical activity and sitting time	Non-government health agency in New South Wales, Australia (n=42)	Yes	Decreased sitting time and increased standing at work
Edmunds et al (35) (2013)	6-month single-arm repeated measures design	Multiple	Physical activity and well-being	Low-active employees from 17 small and medium-sized organizations in the United Kingdom (n = 89)	Yes	Days per week of physical activity
Flannery et al (40) (2012)	3-month quasi-experimental pilot	Multiple	Physical activity and diet	Nursing assistants in a long-term care facility in Baltimore, Maryland (n = 39)	No	None
Healy et al (38) (2013)	4-week non-randomized controlled trial	Single	Physical activity and sitting time	Government office workers in Melbourne, Australia (n = 43)	Yes	Decreased sitting time and increased standing at work
Huang et al (36) (2013)	6-month pilot pretest–posttest	Single	Physical activity, diet, stress management, medication adherence, and alcohol consumption	Manufacturing employees in Taiwan, China, with high blood pressure, cholesterol, or triglycerides (n = 283)	Yes	Increased physical activity readiness-to-change stage
Irvine et al (27) (2011)	1-month randomized controlled trial	Single	Physical activity	Sedentary employees working at a large manufacturing plant in Oregon (n = 228)	Yes	Minutes per day of physical activity and current exercise status
Lemon et al (28) (2014)	24-month cluster randomized trial	Multiple	Physical activity and diet	12 public high schools in Worcester, Massachusetts (n = 782)	No	Not applicable: intervention assessed body mass index, participation in physical activity events, and implementation of physical activity policies at the organization level but not individuals’ physical activity behavior
Mansi et al (29) (2015)	3-month randomized controlled trial	Single	Physical activity	Meat-processing workers in New Zealand (n = 58)	No	None
McEachan et al (30) (2011)	3-month matched-pairs cluster randomized controlled trial	Multiple	Physical activity	44 worksites in United Kingdom (n = 1,260)	No	None
Morgan et al (31) (2012)	3-month randomized controlled trial with wait-list controls	Single	Physical activity and diet	Male shift workers in New Castle, New South Wales (n = 110)	No	None
Pronk et al (39) (2012)	4-week non-randomized time series	Single	Sitting time	Sedentary workers in Minneapolis, Minnesota (n = 34)	Yes	Decreased sitting time at work
Taylor et al (37) (2010)	6-month pre–post pilot study	Single	Physical activity	Small legal business employees in the United States (n = 14)	No	None
Thøgersen- Ntoumani et al (32) (2014)	16-week feasibility trial	Single	Physical activity	Non-academic university employees in the United Kingdom who did not meet current physical activity recommendations (n = 75)	Yes	Time spent active during the week if group-led walk

a Adapted from the Centers for Disease Control and Prevention’s Worksite Health ScoreCard (11).

b Physical activity improvements were reported to be significant (*P* < .05).

Studies targeted a diverse group of worksites, including desk-based (24,26,34,37–39), manufacturing (25,27,29,31,36), and health care worksites (23,33,40). Two studies conducted programs in multiple settings (eg, a university and a bus company) (30,35), 1 study evaluated a public school setting (28), and 1 study evaluated a university setting (32).

Sample sizes varied, both in number of worksites and number of employees enrolled or evaluated. Twelve studies assessed a single worksite (23,26,27,29,31–34,36–39), and 6 studies tested their worksite health promotion program in multiple worksites (24,25,28,30,35,40). Most studies enrolled or evaluated fewer than 100 participants, with 5 evaluating 50 or fewer participants (26,37–40) and 5 evaluating 51 to 100 participants (24,29,32,34,35). Three studies included 101 to 249 participants (23,27,31). Larger studies included those enrolling 250 to 749 participants (25,36) and more than 750 (28,30,33). Multiple countries were represented in the included studies. Only 7 of 18 studies were conducted in the United States (23,25,27,28,37,39,40).

### Intervention component: organizational supports

**Best.** Five studies mentioned tailoring program and education materials to the target population. Three studies conducted employee health risk assessments (HRAs) (25,33,36).

**Better.** All studies positively answered the organizational supports question, “During the past 12 months, did your worksite conduct ongoing evaluations of health promotion programming that use multiple data sources?” Nine studies provided incentives for participating in the intervention study (23,25,26,31,33–35,37,40), and 1 study provided incentives for participating in the wellness program as a whole (39). Eight studies reported involvement and support from management (26,30,33,34,36–39). Seven studies used natural helpers or peer champions as advocates of the program (25,28,30,33,35,37,40).

Five studies used competitions to support employees in making behavior changes (28,30,33,35,40). Flexible work schedule policies were found in 5 studies (29,33,36,39,40). Four studies (24,28,33,38) mentioned a designated health promotion coordinator, but only 1 indicated that the health promotion responsibilities were part of their paid employment and job responsibilities (33), 1 was a designated liaison between the research team and employees (38), 1 was named team leader of the health and well-being group (24), and 1 indicated that a stipend was provided (28). Three studies mentioned a health promotion committee (34,38,39). One study discussed annual health promotion organizational objectives (33). One study mentioned engaging in other health initiatives in the community (33).

**Good.** Program promotion and marketing efforts were found in 9 studies (23,26,27,30,32–34,37,40). Flyers and newsletters were used in 5 studies (23,26,27,32,40), 2 studies used branded programs and logos (30,33), and 1 study offered a program kick-off party (37). Finally, only 1 study mentioned including family members in the worksite health promotion program (23), 1 study mentioned using role modeling to promote behavior change (30), 1 study conducted an employee needs assessment for planning health promotion activities (33), and 1 study included employee health in business objectives/organizational mission statement (37).

### Intervention component: physical activity

**Best.** Nine studies used physical activity programs (other than the use of an exercise facility) (24,25,28,32,33,35–37,40). Among these studies, 6 provided on-site exercise classes (28,33,35–37,40), 5 used walking groups (24,25,28,32,35), 2 used structured physical activity breaks (37,40), and 2 used stretching classes (25,36). Five studies provided other environmental supports for physical activity (24,28,32,33,40). Four studies provided maps of walking routes (24,28,32,33), 1 provided locker rooms (28), and 1 provided video games and DVDs on-site to promote physical activity onsite when the interventionist was not there (40). Four studies provided on-site exercise facilities (28,33,35,40). Two studies subsidized or discounted gym memberships (26,33). One study indicated promoting the use of stairs (33).

Five studies included fitness assessments with follow-up counseling (23,29,34,38,40). Four studies used pedometers (23,29,34,40), and 2 studies used accelerometers (26,38). Follow-up counseling included dietitians and medical professionals (23), occupational health nurses (34), nursing assistants (40), masters-level health coaches (38), and personalized weekly emails about step counts and health information (29).

**Better.** Nine studies incorporated educational classes or seminars addressing physical activity (23,25,27,31,33,34,36,38,40). Among these, 1 study provided an educational class summarizing the health consequences of excessive sitting (38). Eight studies conducted face-to-face sessions (23,25,31,33,34,36,38,40), and 1 study provided online video classes (27).

**Good.** Eleven studies provided employees with information on the benefits of physical activity (23,25,27–31,33–36), and 1 study provided information about the benefits of sitting less (38). One study indicated that physical activity resources were provided but did not specify what types of resources were provided (39).

### Effect of worksite health promotion program on physical activity and alignment with HSC

Table 3 shows the frequency of HSC strategies (organizational supports and physical activity) among the studies included in this review, including a comparison of HSC strategies did and did not result in significant (*P* < .05) improvements in physical activity. All studies targeted physical activity; 15 studies also measured physical activity behavior. Studies that did not measure physical activity behavior measured readiness to change physical activity (36), physical activity quality of life (31), and implementation of physical environment and policy intervention strategies (eg, implementation of a walking group) (28).

**Table 3:** Frequency of Use of CDC’s Health ScoreCard^a^ Strategies by Physical Activity Worksite Health Promotion Programs That Improved Physical Activity and Programs That Did Not

Health ScoreCard Strategy, by Point Value^b^	Overall Use of Strategy, No. of Studies (n = 18)	Use of Strategy Improved Physical Activity, No. of Studies (n = 11)	Use of Strategy Did Not Improve Physical Activity, No. of Studies (n = 7)
**Organizational Supports**			
**Best**			
Conduct employee health risk appraisal/assessment	3	3	0
Tailor program/materials to segments of workforce	5	3	2
**Better**			
Demonstrate organizational commitment of worksite health promotion at all levels of management	8	6	2
Use/combine incentives with other strategies	10	7	3
Use competitions to support behavior changes	5	2	3
Have an active health promotion committee	3	3	0
Have paid health promotion coordinator	4	2	2
Have champion(s) who advocates for program	7	3	4
Set annual health promotion organizational objectives	1	1	0
Conduct ongoing evaluation of program using multiple sources	18	11	7
Provide flexible work scheduling policies	5	3	2
Engage in other community health initiatives	1	1	0
**Good**			
Conduct employee needs /interest assessments	1	1	0
Promote /market worksite health promotion programs to employees	9	6	3
Use role modeling/success stories in marketing materials	1	0	1
Include employee health in business objectives/mission statement	1	0	1
Make programs available to family members	1	1	0

**Physical activity**			
**Best**			
Provided exercise facility on-site	4	2	2
Subsidize/discount cost of exercise facilities	2	2	0
Provide other environmental supports	5	2	3
Post signs that encourage stair use	1	1	0
Provide organized physical activity programs to employees	9	5	4
Provide/subsidize fitness assessments, follow-up counseling, and PA recommendations	5	3	2
**Better**			
Provide PA seminars/classes/workshops	9	7	2
**Good**			
Provide information on the benefits of PA	11	7	4

a Adapted from the Centers for Disease Control and Prevention’s Worksite Health ScoreCard (11).

b The HSC assigns point values of 1, 2, or 3 (1 = good, 2 = better, and 3 = best) to indicate the level of effect each strategy has on the associated health topic or outcome and the strength of evidence for this effect.

Eleven studies showed significant improvements (23,25–27,32–36,38,39). Examples of physical activity improvements include the following: general increased physical activity (27,32,33), decreased sitting time (26,38,39), increased steps (23,34), increased standing time (26,38), stretching/flexibility (25), days of physical activity (35), minutes of physical activity (27), active travel (33), increased activity at work (33), and increased readiness to change (36).

**Organizational supports.** The most common HSC organizational supports strategies that produced significant physical activity improvements were leadership support, incentives, and marketing. Among the 8 studies demonstrating leadership support, 6 produced improvements in physical activity or sitting behaviors (26,33,34,36,38,39). Among the 8 studies that indicated using promotion and marketing, 5 produced significant physical activity improvements (23,26,27,32,33). Among the 10 studies that used or combined incentives with other strategies, 7 produced significant results (23,25,26,33–35,39).

In a comparison of the organizational supports HSC strategies that resulted in physical activity improvements and those that did not result in improvements, 5 strategies that resulted in improvements emerged: leadership support, incentives, employee HRAs, active health promotion committees, and marketing (Table 3). All 3 studies that had an active health promotion committee (34,38,39) and that conducted employee HRAs had significant improvements (25,33,36).

**Physical activity.** Of the 11 studies with significant physical activity improvements, the 4 most common physical activity HSC strategies were 1) providing information on the benefits of physical activity; 2) providing physical activity classes, seminars, or workshops; 3) providing organized physical activity programs; and 4) providing or subsidizing fitness assessments, follow-up counseling, and physical activity recommendations. Seven of 11 studies that provided information on the benefits of physical activity had significant improvements (23,25,27,29,34–36). However, 4 of the 7 studies that did not produce significant improvements also included information on physical activity benefits (29–31,40).

Among the 9 studies that provided physical activity seminars, workshops or classes, 7 had significant improvements (23,25,27,33,34,36,38). Among the 9 studies that provided organized individual or group physical activity programs, 5 had significant improvements (25,32,33,35,36). However, 4 of the 7 studies that did not produce significant improvements also provided organized programs to employees (24,28,37,40).

In a comparison of the physical activity HSC strategies that resulted in physical activity improvements and those that did not result in improvements, 2 components that resulted in significant improvements emerged: subsidizing or discounting the cost of exercise facilities and providing physical activity seminars, classes, and workshops (Table 3).

## Discussion

The worksite provides an important setting for addressing the adult obesity epidemic and improving the health of the working population by targeting physical activity. Research consistently demonstrates a relationship between worksite health promotion programs and improvements in health behaviors of employees. This scoping review highlights evidence-based worksite health promotion strategies promoted through the CDC’s HSC. The HSC organizational supports strategies of leadership support (better), incentives (better), employee HRAs (best), and active health promotion committees (better) and the physical activity strategies of subsidizing or discounting the cost of exercise facilities (best) and providing physical activity seminars, classes, and workshops (better) showed the greatest impact on physical activity improvement.

Each of these findings can be used to guide recommendations for future worksite health promotion research and practice. All notable strategies were categorized, by using a point system, as either better or best, reinforcing the importance of these components. Using the HSC to synthesize these findings also demonstrates the need to promote the use of a framework for worksite health promotion planning and evaluation.

Although improvements were found among programs using leadership support, less than half of the studies used leadership support, and even fewer studies implemented policies, such as flexible scheduling or paid breaks, that support the worksite health promotion program in encouraging employees to engage in physical activity. Leadership support increases worksite health promotion participation, reduces job stress, and improves health behavior (41–44). These studies demonstrate areas of focus for future worksite health promotion programs. Leadership support and other key HSC organizational supports strategies should be further explored and assessed, including the strategies that did not have a significant effect on physical activity, such as using competitions, having a paid health promotion coordinator, having champions advocate for the program, using role modeling and success stories in marketing, and including health in business objectives, to further understand the importance of creating a supportive worksite health promotion environment for employees.

Similarly, although the use of health champions was common in our studies, the lack of physical activity improvement raises questions about the definition of a health champion, the types of incentives health champions are offered, and the relationship between health champions and coworkers. Previous studies found that some of the best strategies for improving employee health include using leaders as mentors and champions of workplace values (45), creating a corporate culture that acknowledges the importance of employee health and offering participation-friendly corporate policies and physical environments (46). Future worksite health promotion research should explore the effectiveness of organizational supports strategies and their effect on both health-related outcomes and job satisfaction outcomes.

We observed a trend among interventions demonstrating improvements in physical activity behaviors and the use of activity trackers. Although the use of activity trackers is not included in the HSC, 9 of the 11 studies that showed improvements in physical activity behaviors or activity at work used pedometers (23,24,29,32–35) or accelerometers (26,38). Therefore, the inclusion of a pedometer or other activity tracker, other than or in addition to self-report, could be a key contributing factor to motivating participants to increase their activity levels. The addition of an activity tracker strategy to the HSC could be considered.

Finally, the role of organizational supports may strongly influence program effectiveness through incentives, HRAs, leadership support, health promotion committees, and marketing. Previous studies identified the positive impact of organizational supports on employee well-being and work engagement (47,48). We also found that organizational supports strategies had an impact on physical activity. A greater number of organizational supports strategies (n = 3) than physical activity strategies (n = 2) produced significant physical activity improvements. This finding reinforces the importance of creating a supportive workplace environment and culture when targeting physical activity behaviors. Future physical activity worksite health promotion programs should promote and evaluate organizational supports strategies in addition to physical activity outcomes.

Studies that incorporate needs assessments to gauge employee preferences for health topics, program components, and availability are lacking. A mismatch between employee interests, schedules, and availability and the worksite health promotion program may limit program participation (49). Although many programs provided a rationale for the health topic of interest, only 1 study directly collected feedback on employee preferences before launching the program. Asking employees about their preferences could provide critical insight into motivating employees to participate, resulting in a more effective program.

Lack of time is a consistent barrier to making health behavior changes among working adults (50). Providing programs at the worksite provides a realistic strategy for overcoming this barrier while also reaching overweight or obese working adults. The interventions assessed in this study demonstrate the potential effectiveness of worksite health promotion programs in helping adults improve physical activity behaviors, which can result in improvements in body mass index and other health-related outcomes, such as blood pressure and cholesterol levels. The workplace can also promote sustainability of behavior changes because working adults spend consistent, substantial amounts of time there.

One potential limitation of this review is the methodology of the literature search, which may have excluded some studies in the initial screening. Publication bias might also have been a limitation, given that all studies included in the review reported positive program outcomes. Additionally, the methods outlined by the authors in their articles were used to complete portions of the HSC. If intervention methods were not fully described in an article, then they would not have been reported in our review.

Studies included in our review had some methodological limitations, which may have affected some of our conclusions. For example, some studies lacked randomization and use of a control group, which may have limited their internal validity. Many studies enrolled fewer than 100 participants, which likely limited the generalizability to larger worksites. Selection bias may have also been present since employees who are motivated and healthy may be more likely to participate in worksite health promotion program than employees who are less motivated and less healthy. The length of worksite health promotion programs and follow-up period also varied; some programs may not have been in operation long enough to realize program effects.

Despite these limitations, our review used a novel approach to identify key strategies implemented by effective worksite health promotion programs. Organizational-level policies such as incentives, HRAs, health promotion committees, and demonstrations of leadership support may be more likely than other policies to improve employee physical activity and, potentially, productivity. The inclusion of the HSC provided insight into program components that affect physical activity behaviors. Our results could be used to inform workplace wellness committees and decision makers in occupational settings about policies and organizational supports that facilitate healthy behavior changes, especially for physical activity. Future research should focus on using the HSC framework to assess worksite health promotion programs and finding effective strategies for motivating workers to stay engaged in worksite health promotion programs.
